# Effects of the ß2-Adrenoceptor Agonist, Albuterol, in a Mouse Model of Anti-MuSK Myasthenia Gravis

**DOI:** 10.1371/journal.pone.0087840

**Published:** 2014-02-05

**Authors:** Nazanin Ghazanfari, Marco Morsch, Nigel Tse, Stephen W. Reddel, William D. Phillips

**Affiliations:** 1 Physiology and Bosch Institute, University of Sydney, Sydney, New South Wales, Australia; 2 Department of Molecular Medicine, Concord Hospital, Concord, New South Wales, Australia; Georgia Regents University, United States of America

## Abstract

The β2-adrenergic receptor agonist, albuterol, has been reported beneficial in treating several forms of congenital myasthenia. Here, for the first time, we examined the potential benefit of albuterol in a mouse model of anti-Muscle Specific Kinase (MuSK) myasthenia gravis. Mice received 15 daily injections of IgG from anti-MuSK positive patients, which resulted in whole-body weakness. At neuromuscular junctions in the tibialis anterior and diaphragm muscles the autoantibodies caused loss of postsynaptic acetylcholine receptors, and reduced the amplitudes of the endplate potential and spontaneous miniature endplate potential in the diaphragm muscle. Treatment with albuterol (8 mg/kg/day) during the two-week anti-MuSK injection series reduced the degree of weakness and weight loss, compared to vehicle-treated mice. However, the compound muscle action potential recorded from the gastrocnemius muscle displayed a decremental response in anti-MuSK-injected mice whether treated with albuterol or vehicle. Ongoing albuterol treatment did not increase endplate potential amplitudes compared to vehicle-treated mice nor did it prevent the loss of acetylcholine receptors from motor endplates. On the other hand, albuterol treatment significantly reduced the degree of fragmentation of endplate acetylcholine receptor clusters and increased the extent to which the remaining receptor clusters were covered by synaptophysin-stained nerve terminals. The results provide the first evidence that short-term albuterol treatment can ameliorate weakness in a robust mouse model of anti-MuSK myasthenia gravis. The results also demonstrate that it is possible for albuterol treatment to reduce whole-body weakness without necessarily reversing myasthenic impairment to the structure and function of the neuromuscular junction.

## Introduction

Most cases of autoimmune myasthenia gravis (MG) are caused by autoantibodies against the nicotinic acetylcholine receptor (AChR). Anti-AChR IgG reduces the efficacy of synaptic transmission at the neuromuscular junction (NMJ) by blocking AChR channels, accelerating AChR degradation and activating complement [1]. A subset of MG patients possess autoantibodies against muscle-specific kinase (MuSK) [2], [3] or its partner protein, low-density lipoprotein receptor-related protein 4 (LRP4) [4], [5], instead of AChR autoantibodies. MuSK is a postsynaptic transmembrane tyrosine kinase that is essential for the formation and stabilization of AChR clusters at the developing NMJ [6]. Neural agrin, a proteoglycan secreted by motor axons, binds to LRP-4 triggering the assembly of the membrane-spanning MuSK protein complex [7], [8]. A critical step in activation of MuSK kinase is the formation of a heterotetramer of two molecules of MuSK and two molecules of the intracellular adaptor protein Dok-7 [9], [10]. Once activated, the MuSK complex initiates multiple signaling events that coordinate the assembly and stabilization of the developing postsynaptic membrane proteins [11], [12].

Drugs that activate the β-adrenoceptor offer therapeutic benefit in certain forms of congenital myasthenia syndrome that share some features with anti-MuSK MG. Ephedrine has been in clinical use for myasthenic disorders at least since the 1930 s [13]. Open label studies have reported ephedrine and another β-adrenoceptor-activating drug, albuterol (also known as salbutamol), beneficial in treating congenital myasthenias involving mutations that cause deficits in Dok-7, AChR (ε-subunit) or ColQ and synaptic acetylcholinesterase [14], [15], [16], [17], [18], [19], [20]. In a mouse model of slow channel syndrome the effects of albuterol and ephedrine were equivocal [21]. These observations in patients prompted us to test, for the first time the efficacy of albuterol in a vehicle-controlled animal model of MG.

Current treatment options for the anti-MuSK form of MG have significant drawbacks. Clinical series of anti-MuSK MG patients have confirmed the utility of antibody based therapies (plasmapheresis being more effective than IVIG) and immunosuppression with corticosteroids, antiproliferative agents and rituximab. However these treatments are associated with significant risks and many are expensive [22], [23], [24]. Cholinesterase inhibitors such as pyridostigmine are frequently prescribed for MG patients [25], [26], [27]. By inhibiting acetylcholinesterase (AChE) these drugs prolong the actions of acetylcholine in the synaptic cleft and increase the endplate potential (EPP) amplitude. However, in some reports pyridostigmine was ineffective or even harmful in anti-MuSK MG patients [28], [29], [30]. In our mouse passive IgG transfer model of anti-MuSK MG pyridostigmine provided no benefit, but rather exacerbated the loss of endplate AChRs, further reduced synaptic function and triggered myasthenic weakness in some mice [31]. Together these findings indicate the need for additional therapeutic options for the anti-MuSK form of MG.

Autoantibodies specific for MuSK seem to cause MG by disrupting MuSK-dependent maintenance of the NMJ. Active immunization of animals with MuSK, or passive transfer of IgG from anti-MuSK-positive MG patients resulted in whole-body weakness that was associated with impaired neuromuscular transmission [32], [33], [34], [35], [36],[37],[38]. MuSK autoantibodies from patients appear to be mainly of the IgG4 subclass and endplate pathology in the experimental animals did not seem to involve T-lymphocyte- or complement-mediated damage [3], [36], [39], [40]. Rather, MuSK autoantibodies are suspected to disrupt the physiological role of MuSK in maintenance of the NMJ. Cell culture experiments showed that bivalent anti-MuSK IgG can trigger activation of MuSK, while monovalent anti-MuSK can inhibit the activation of MuSK by neural agrin [32], [41], [42], [43], [44]. When added to heterologous cells, anti-MuSK-positive patient IgG caused internalization of MuSK-GFP [41]. Daily injections of anti-MuSK-positive patient IgG into mice depleted MuSK from the motor endplate [41]. In contrast, Viegas and colleagues did not find any reduction in endplate MuSK in mice actively immunized against MuSK [37]. Thus, MuSK autoantibodies might interfere with MuSK signaling at the NMJ in several different ways, perhaps depending upon the IgG isoform and the precise epitope target.

In common with the Dok-7 congenital myasthenia, anti-MuSK MG is thought to interfere with MuSK signaling in the postsynaptic membrane. Ephedrine is a restricted drug, being a precursor of amphetamine while albuterol, a selective β2 adrenergic agonist, is widely used as a bronchodilator [45]. Hence we have examined the effects of albuterol on clinical presentation, the NMJs and the muscle fibers in a mouse model of anti-MuSK MG. One hypothesis was that impaired MuSK-mediated AChR clustering might be compensated by activation of muscle β2-adrenoceptors [18]. In mice injected with anti-MuSK-positive patient IgG continuous albuterol treatment reduced muscle weakness and body-weight loss. Surprisingly, albuterol did not inhibit the anti-MuSK-induced loss of endplate AChRs nor did it enhance EPP amplitudes. These findings suggest that it is possible for albuterol to improve strength (at least in one model of anti-MuSK MG) without necessarily reversing the myasthenic impairment of neuromuscular transmission.

## Methods

### Ethical Statement

All mouse experiments described in this paper were conducted at the University of Sydney with the approval of The University of Sydney Animal Ethics Committee in accordance with the New South Wales Government Animal Research Act 1985, associated regulations (2005) and the Australian Code of Practice for the Care and Use of Animals for Scientific Purposes, 7th edition (National Health and Medical Research Council, 2004). In relation to the collection of plasma, informed, written consent was obtained from patients in accordance with the Declaration of Helsinki (5th revision, 2004). The project was approved by the Human Research Ethics Committee of the Sydney South West Area Health Service.

### Passive IgG Transfer Experiments

Female C57BL/6J mice (6-week-old from Animal Resources Centre, Western Australia) received 15 daily intraperitoneal (I.P.) injections of anti-MuSK-positive patient IgG (25 mg filter-sterilized in PBS) as previously described [33], [38], [41]. The IgG was collected from therapeutic plasma exchange of patient #7 when at Myasthenia Gravis Foundation of America (MGFA) grade 3B. On day 15 mice were killed with pentobarbitone (30 mg I.P.; Cenvet Australia). A single I.P. injection of cyclophosphamide monohydrate (300 mg/kg; Sigma, St Louis, MO; 10 mg/ml in 0.9% NaCl) was given 24 h after the first IgG injection to suppress any active immune response to the human proteins [46].

### Drug Treatment

Mice were treated with albuterol (2, 4 or 8 mg/kg/day) infused steadily for 2-weeks via an Alzet minipump (model 1002; Durect Corporation, Cupertino, CA) that was implanted beneath the skin of the mid back on day 0 of the IgG injections. Albuterol sulfate (Sigma) was dissolved in sterile water (vehicle). To implant the pump, mice were anaesthetized with 1–3% isoflurane/oxygen. Mice received buprenorphine (0.03 mg/kg subcutaneously; Reckitt Benckiser, Australia) for analgesia at the completion of surgery and 24 h later, as previously described [31].

### Assessment of Weakness and Weight

Mice were inspected daily for general health and fatigue and were weighed and graded for muscle weakness before and after an exercise regime that involved the mouse walking backwards 12 times over a wire cage grid [33], [47]. Grade 1 weakness refers to a mouse that behaves normally at rest but displays myasthenic symptoms after exercise including: body prone, chin down, flaccid tail, and forelimb weakness. Grade 2 refers to a mouse that displayed the same fatigued/immobile behavior before the exercise. For comparison of the 8 mg/kg/day albuterol-treated versus vehicle-treated mice a subset of the mice (2/4 from each treatment group) were video recorded on day 14 (and/or day 15). The videos were independently evaluated for clinical grade by another researcher who was unfamiliar with the mice and was blind to treatment assignment. Their results were found to be concordant with the primary grading. For ethical reasons, mice were killed with pentobarbitone (30 mg I.P.) if they reached grade 2 weakness. Mice were group-housed in filter-top cages with wire-grid lids (17×34×13 cm WxLxH), had free access to water and YSF Brand rat and mouse chow. We, and others have previously observed that progressive weight loss in the anti-MuSK MG model coincides with clinical weakness [33], [36].

### Electrophysiology

Repetitive stimulation of the sciatic nerve and compound muscle action potential (CMAP) recordings were performed on mice anaesthetized with isoflurane/oxygen as previously described [38]. Briefly, the gastrocnemius muscle was prepared with abrasive skin prepping gel (Nuprep, D.O. Weaver & Co, Aurora, USA). Two custom-made single monopolar 3 mm recording electrodes were glued to the surface of the skin: one over the dorsal aspect of the gastrocnemius muscle and the second electrode at the ankle of the same hind limb. Electrolyte gel (VIASYS Healthcare, Madison, USA) was applied directly at the electrode sites. Stimulation of the sciatic nerve was accomplished via a 4 mm incision in the sciatic notch and by placing the nerve on a custom-made silver hook electrode (0.6 mm diameter).

Spontaneous miniature endplate potential (mEPP) and evoked endplate potential (EPP) recordings were made from phrenic nerve-hemidiaphragm preparations at physiological calcium levels (1 mM MgCl_2_, 2 mM CaCl_2_) as previously described [38]. Contraction was blocked using the muscle sodium channel blocker µ-conotoxin GIIIb (1 µM µCTX, Peptide Institute, Japan). EPP and mEPP recordings were performed 30–60 min after the diaphragm was pinned out in the bath solution. Spontaneous mEPP amplitudes were normalized to a resting potential of −80 mV. EPP amplitudes were normalized to −80 mV and then corrected for non-linear summation [48]. The EPP amplitude was taken as the average value of a train of 40 stimuli at 1/sec. Quantal content was calculated by dividing the normalized and corrected EPP amplitudes by the normalized mEPP amplitude for each muscle fiber.

### Immunostaining and Morphometric Analysis of Motor Endplates

To compare AChR staining intensities, transverse sections (12 µm) of the tibialis anterior and diaphragm muscles were fixed for 15 min in 2% paraformaldehyde/phosphate buffered saline (PBS) at room temperature. Sections were blocked for 1 h in 2% bovine serum albumin (BSA)/PBS and then incubated with Alexa488-α-bungarotoxin (Alexa488-BGT; Molecular Probes, 1∶200) for 1 h at room temperature. Slides were washed three times with PBS between every step. For comparisons of staining intensities, all slides were stained in the same batch, and confocal optical sections of endplates were collected in the same imaging session using fixed gain and black level settings (Zeiss LSM510 Meta confocal microscope). ImageJ 1.31 v software (National Institutes of Health; Bethesda, MD; http:rsb.info.nih.gov/ij) was used to measure the fluorescence intensities.

The area of NMJ specializations was assessed using longitudinal sections of the diaphragm muscle (20 µm). Sections were fixed as described above and nerve terminals were labeled using a cocktail of rabbit anti-neurofilament antibody (1∶8000; Sigma) and rabbit anti-synaptophysin antibody (1∶200; Dako Australia) overnight at 4°C. After washing three times with PBS, sections were incubated with fluoresceine isothiocyanate (FITC)-conjugated donkey anti-rabbit IgG (1∶250; Jackson ImmunoResearch) and Alexa555-α-bungarotoxin (Alexa555-BGT; Molecular Probes, 1∶200) for 1 h at room temperature. Image stacks (for maximum-intensity Z-projection images) were collected enface to the endplate (Zeiss LSM510Meta confocal microscope) in the same imaging session. Metamorph software (Molecular Devices Inc., CA, USA) was used to measure the area and intensity of the postsynaptic AChR cluster and presynaptic nerve terminal staining. The co-localization plug-in of the Metamorph program was used to determine the area of overlap (pixel by pixel) [38].

Photomicrographs of endplates from anti-MuSK-injected mice often revealed dim staining. To improve visibility the brightness was increased in the final figures. Changes in brightness were applied in equal proportion to the control and experimental panels of the figure in question.

The cross-sectional areas of muscle fibers were measured from transverse sections (12 µm) of the tibialis anterior and diaphragm muscles. Sections were fixed for 15 min in 4% paraformaldehyde/PBS and pre-incubated for 1 h in 10% BSA/PBS. Rabbit anti-laminin antibody (1∶100; Sigma overnight at 4°C) followed by FITC-conjugated donkey anti-rabbit IgG (1∶250; Jackson ImmunoResearch) were used to label the basement membrane. The laminin-stained basement membrane was used to frame the cross-sectional area of each muscle fiber, which was then measured using ImageJ software.

### Statistics

Graphpad Prism (GraphPad Software, CA, USA) was used for statistics. Comparison of two groups (albuterol-treated versus vehicle-treated mice) was by unpaired, two-tailed Student’s t-test where n was the number of mice in the treatment group. The cross-sectional area of fibers in the tibialis anterior muscle did not follow a normal distribution. Therefore one-tailed Mann-Whitney tests (n = number of mice per group) were used to test the hypotheses: 1/that anti-MuSK IgG injections would reduce fiber cross-sectional area, and 2/that albuterol treatment would increase cross-sectional area compared to vehicle treatment. One-way ANOVA with Bonferroni’s multiple comparison post-test was used for comparison of multiple treatment groups. P<0.05 was considered statistically significant.

## Results

### Albuterol Treatment Reduces Weakness and Weight Loss in Anti-MuSK-injected Mice

**Figure 1:**
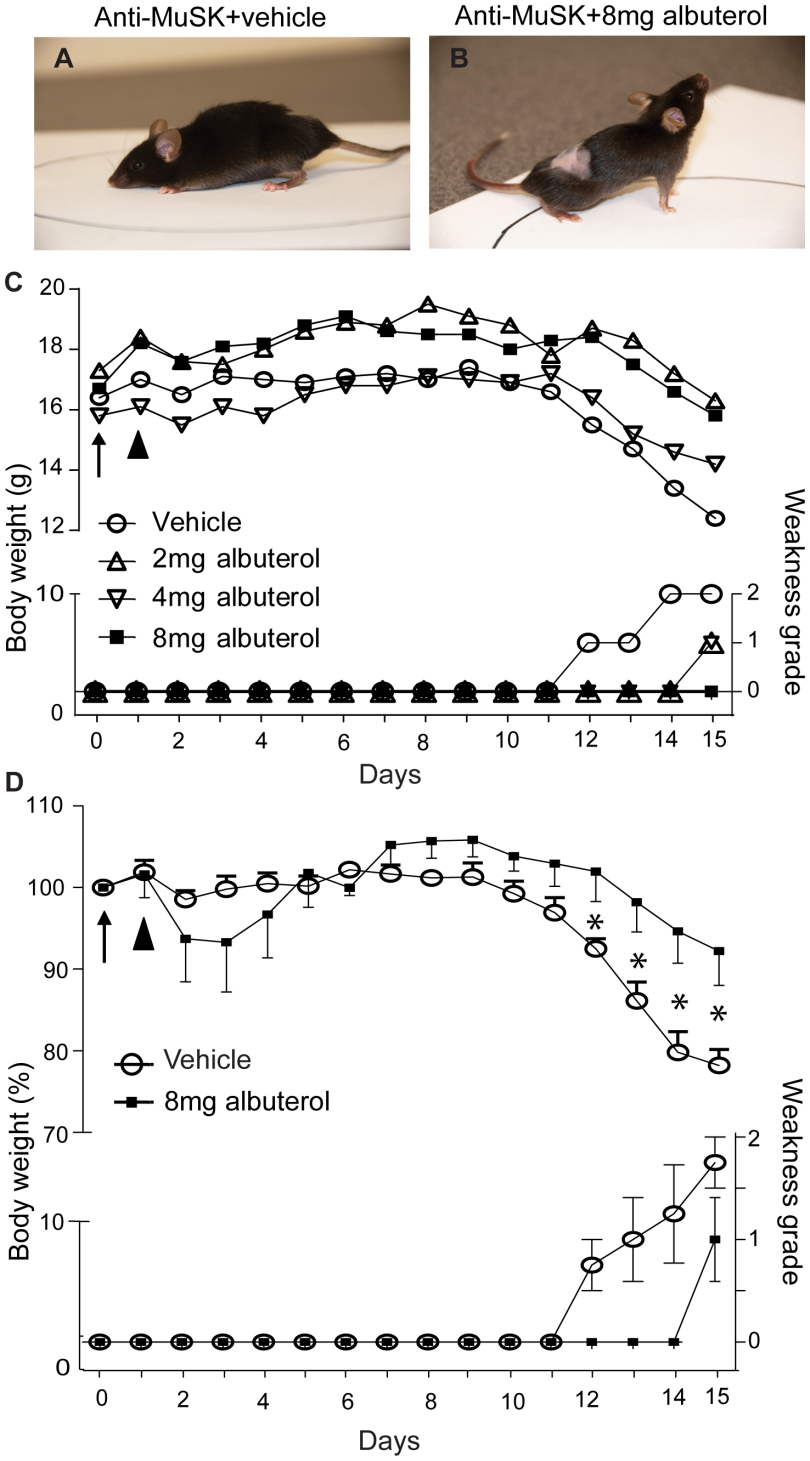
Effects of anti-MuSK IgG and albuterol on muscle weakness and body weight. Mice received 15 daily intraperitoneal injections of anti-MuSK-positive patient IgG. Albuterol was delivered continuously via a subcutaneous minipump. (**A**) Example of typical whole-body weakness in a mouse on day 15 of the anti-MuSK IgG injection series (chin down, flaccid tail, and limb weakness). This mouse was treated with vehicle only. (**B**) A physically active mouse on day 15 of the anti-MuSK IgG injection series with albuterol treatment (8 mg/kg/day). (**C**) Pilot dosage trial. Body-weight and weakness grading traces are shown for single mice that received daily injections of anti-MuSK IgG and were treated with 2, 4 or 8 mg/kg/day albuterol, or vehicle. Body weights are shown on the left ordinate while weakness grades are indicated on the lower right ordinate. A minipump delivering either albuterol or vehicle was implanted subcutaneously on day 0 (arrow). The arrowhead indicates a single cyclophosphamide injection to suppress an active immune response to the human IgG. (**D**) Normalized body weights and weakness grades for mice receiving daily injections of anti-MuSK IgG and treatment with 8 mg/kg/day albuterol (filled squares) or vehicle (open circles). Error bars in panel D represent the mean ± SEM for n = 4 mice in each treatment group (*P<0.05, unpaired Student’s t test).

Mice received daily injections of anti-MuSK-positive patient IgG (25 mg/day I.P.). This led to weight loss and the onset of whole-body weakness between days 12 and 15 of the injection series, consistent with previous findings [31], [33], [38], [41]. Mice were treated with albuterol, delivered systemically via an osmotic minipump, during the two-week IgG injection series. Mice treated with vehicle-only became weak from day 12 onward (Fig. 1A, and open circles in Fig. 1C and 1D). In a pilot experiment single mice treated with 2, 4 or 8 mg/kg/day albuterol showed delayed and reduced whole-body weakness (Fig. 1C). In an expanded group of 4 mice injected with anti-MuSK-positive IgG, treatment with 8 mg/kg/day albuterol significantly reduced the degree of weight loss and weakness compared to vehicle-treated mice (Fig. 1D, compare filled squares with open circles for days 12–15).

### Influence of Anti-MuSK IgG and Albuterol upon NMJ Structure

**Figure 2:**
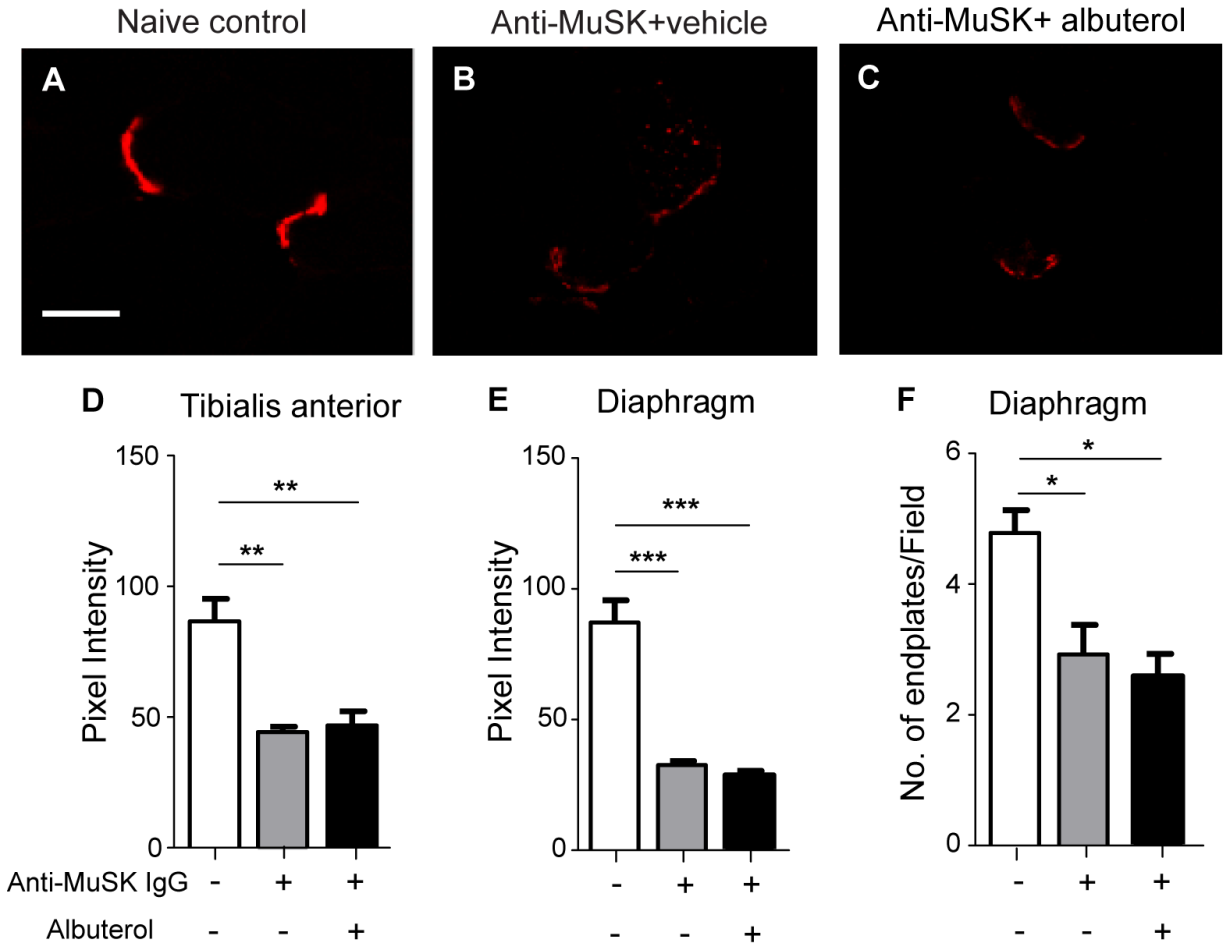
Effects of anti-MuSK IgG and albuterol treatment upon AChR staining intensity. Transverse sections of the diaphragm and tibialis anterior muscles collected on day 15 of the IgG injection series were stained for motor endplate AChR with Alexa488-α-bungarotoxin. (**A**) Typical bright, crescent-shaped AChR staining of two endplates in the diaphragm muscle of a healthy naive mouse (no treatment). Scale bar = 20 µm. (**B–C**) Dim AChR staining of endplates from mice that received injections of anti-MuSK IgG and were treated with either vehicle (B) or 8 mg/kg/day albuterol (C). (**D**) Intensity of endplate staining for AChR (mean pixel intensity) in the tibialis anterior muscle of naive mice and mice that received 15 daily injections of anti-MuSK IgG, with or without albuterol treatment (8 mg/kg/day). (**E**) Intensity of endplate AChR staining in the diaphragm muscle of the same mice. (**F**) Counts of the number of AChR-stained endplates per microscope field in the diaphragm muscle of healthy naive mice, or mice injected with anti-MuSK IgG and treated with albuterol (8 mg/kg/day), or vehicle. Counts were made by an operator who was blind to the treatment group of the photomicrographs. Data in D, E and F represent the mean ± SEM for n = 3 mice (*P<0.05, **P<0.01, ***P<0.001; one-way ANOVA with Bonferroni’s multiple comparison post-test).

To assess the degree of AChR loss from motor endplates we compared the intensity of endplate AChR staining in mice that received injections of anti-MuSK IgG, with and without albuterol treatment. In transverse sections of healthy naive mice, endplates appeared as intensely fluorescent crescents of AChR staining (Fig. 2A). After 15 daily injections of anti-MuSK IgG, endplate AChR staining was relatively dim (Fig. 2B). As expected, the average intensity of endplate staining for AChR was reduced in both the tibialis anterior and diaphragm muscles when compared to healthy naive mice (Figs. 2D and 2E compare grey bar with open bar [33], [38], [41]). In mice injected with anti-MuSK IgG, treatment with albuterol (8 mg/kg/day) did not increase the intensity of endplate AChR staining compared to vehicle-treated mice (Fig. 2C, D & E compare black and grey bars). Blind counts in the diaphragm muscle of mice injected with anti-MuSK IgG revealed a reduction in the number of AChR-stained endplates per microscope field, consistent with complete dismantling of many endplates [41]. Albuterol treatment did not prevent this (Fig. 2F).

**Figure 3:**
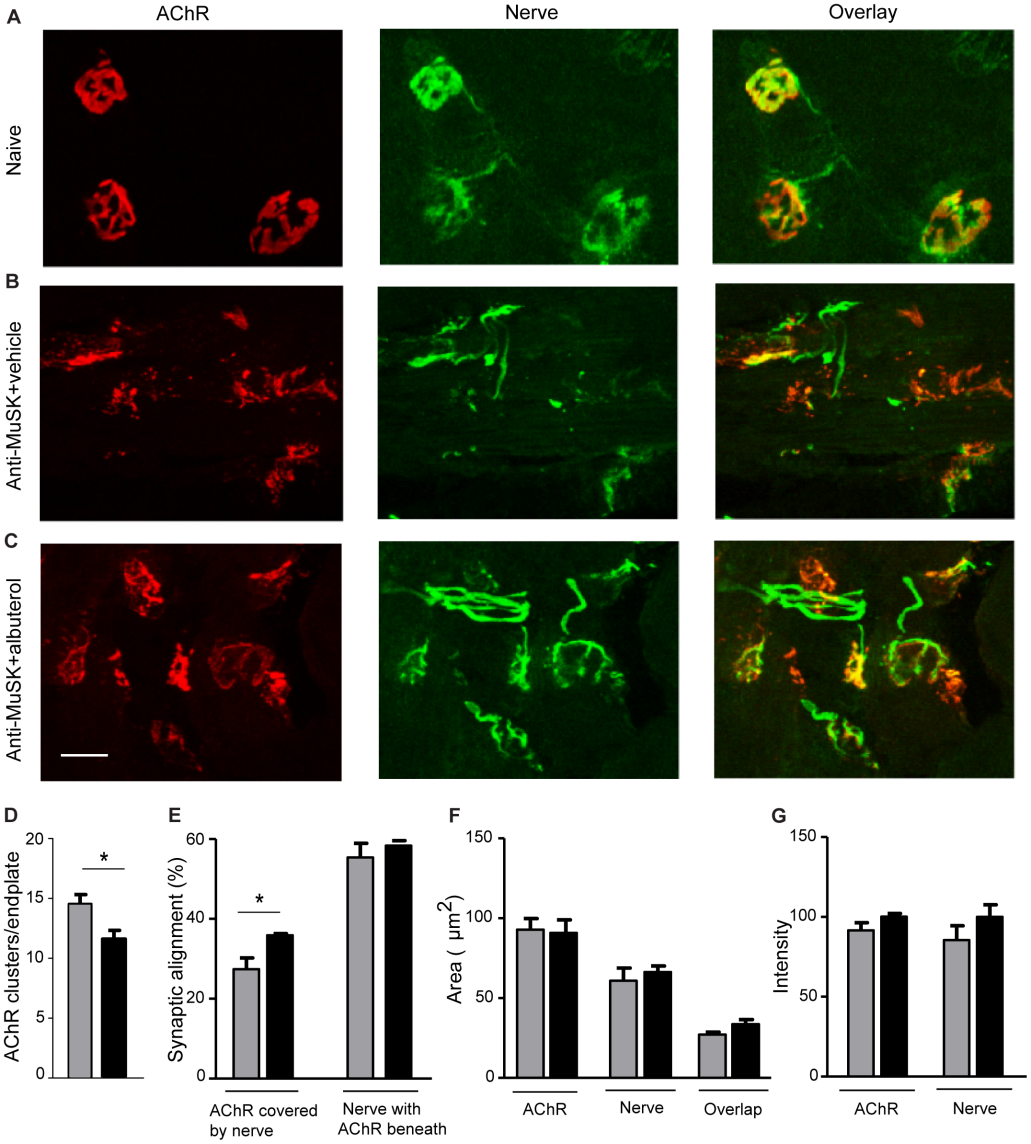
Longitudinal enface images of motor endplates in the diaphragm muscle of mice after injections of anti-MuSK IgG and albuterol treatment. (**A**) Three exemplar NMJs in a healthy naive mouse, double labeled for AChR (red) and nerve (synaptophysin and neurofilament; green), illustrating typical large, pretzel-shaped AChR plaques each covered by nerve terminal staining. (**B**) NMJs from a mouse that received injections of anti-MuSK IgG and treatment with vehicle. Endplate AChR staining was weak and AChRs were fragmented into multiple small AChR clusters. (**C**) NMJs from a mouse that received injections of anti-MuSK IgG and treatment with albuterol (8 mg/kg/day). AChR clusters were less fragmented compared to those of vehicle-treated mice. Some of the nerve terminals appeared better aligned with AChRs. Scale bar is 20 µm. (**D–G**) Quantitative comparison of endplates from mice treated with either 8 mg/kg/day albuterol (black bars) or vehicle (grey bars) at day 15 of the anti-MuSK IgG injection series. (**D**) Average number of AChR clusters per endplate (**E**) Percentage of endplate AChRs covered by nerve and percentage of endplate nerve terminal staining with AChRs clustered beneath it. (**F**) Area of synaptic specialization at the endplate: AChR, nerve and AChR-nerve overlap. (**G**) Relative intensity of endplate AChR staining and nerve staining. Bars represent the mean SEM for n = 3 mice in each treatment group (*P<0.05).

Enface images of the motor endplates from healthy diaphragm muscles typically reveal pretzel-shaped AChR plaques, each covered by a branched nerve terminal (Fig. 3A). Key changes to NMJ structure following injections of anti-MuSK IgG were dimmer AChR staining and fragmentation of the endplate AChR plaque (Fig. 3B; [38], [41]). Endplates of healthy mice generally consist of between 1–3 discrete AChR clusters but MuSK autoantibodies often caused the endplate to fragment into >10 small AChR clusters ([34]; unpublished data). Endplate AChRs of mice injected with anti-MuSK IgG remained fragmented after albuterol-treatment, but were significantly less fragmented than vehicle-treated mice (Fig. 3C, D). In our previous work 51±3% of the AChRs at healthy endplates were covered by nerve staining and this was reduced to 36±4% after a series of injections of anti-MuSK-positive IgG [31]. In the present study, 27±3% of endplate AChRs were covered by nerve staining after the anti-MuSK injections with vehicle treatment. Treatment with albuterol increased this to 36±0.4%, suggesting slightly better retention of nerve terminals by the AChR clusters that remained (Fig. 3C, E). However, albuterol treatment did not significantly increase either the absolute area or the relative intensity of AChR or nerve staining at endplates (Fig. 3F & G).

### Influence of Anti-MuSK IgG and Albuterol upon NMJ Function

**Figure 4:**
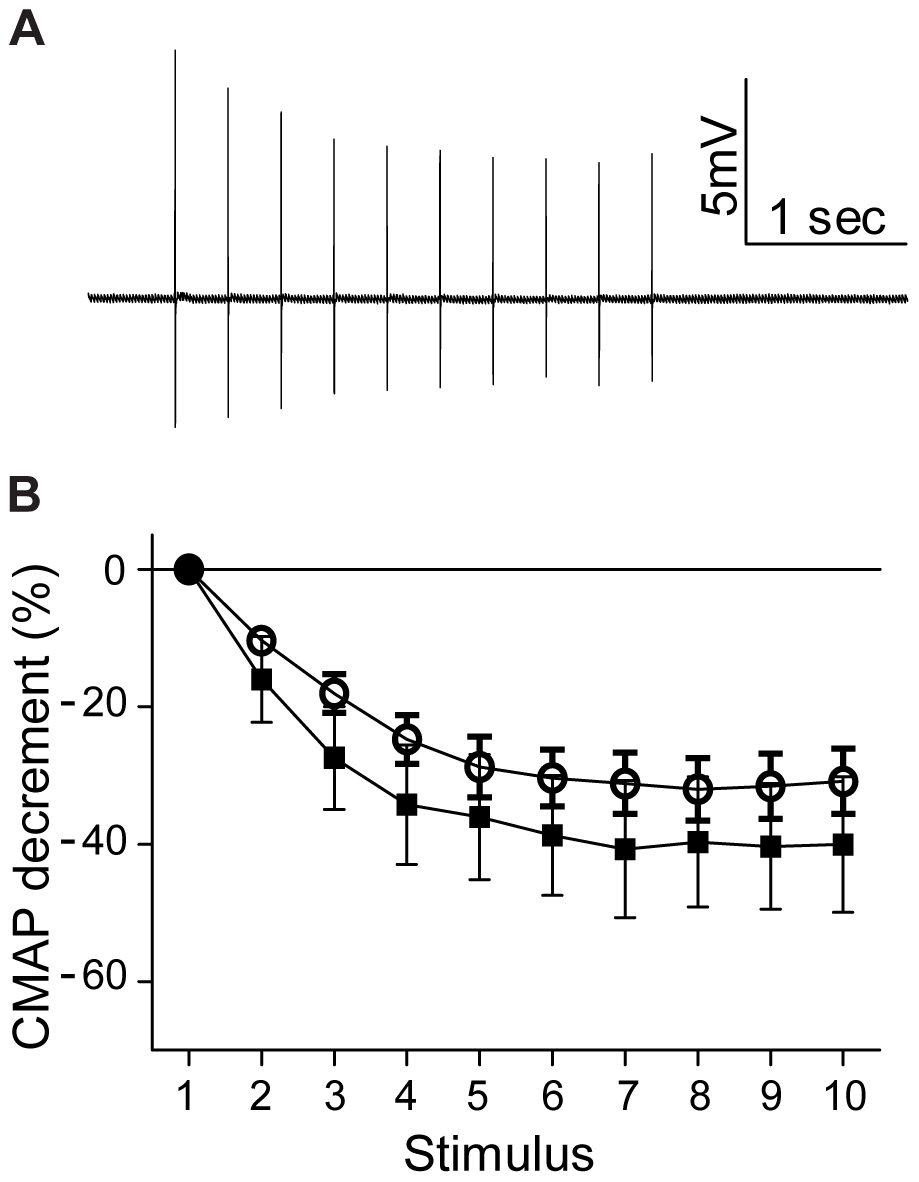
Albuterol treatment did not prevent neuromuscular failure in mice injected with anti-MuSK IgG. (**A**) A representative compound muscle action potential (CMAP) trace recorded from the gastrocnemius muscle of a mouse after 15 daily injections of anti-MuSK IgG. The amplitude is seen to decline during repetitive stimulation of the sciatic nerve (3 stimuli per second). (**B**) Average decrement in CMAP amplitude for myasthenic mice treated with either albuterol (8 mg/kg/day; filled squares) or vehicle (open circles) on day 15 the anti-MuSK IgG injection series. The mean amplitude of the first CMAP of the train was 9.6±2.7 mV for mice injected with anti-MuSK IgG and treated with albuterol compared to 13.5±2.1 mV for mice injected with anti-MuSK IgG and treated with vehicle (P>0.05). Data represent the mean ± SEM for n = 3–4 mice in each treatment group.

In healthy muscles of anaesthetized mice repetitive stimulation of the nerve produces compound muscle action potentials (CMAPs) of consistently high amplitude. After 15 daily injections of anti-MuSK IgG, CMAP recordings from the gastrocnemius muscle revealed a decrementing response during repetitive stimulation of the sciatic nerve (3 stimuli per sec), suggesting impaired neuromuscular transmission (Fig. 4A & B open circles). When compared to vehicle-treated mice, albuterol improved neither the average amplitude of the CMAP nor the CMAP decrement during a train of stimuli (Fig. 4B).

**Figure 5:**
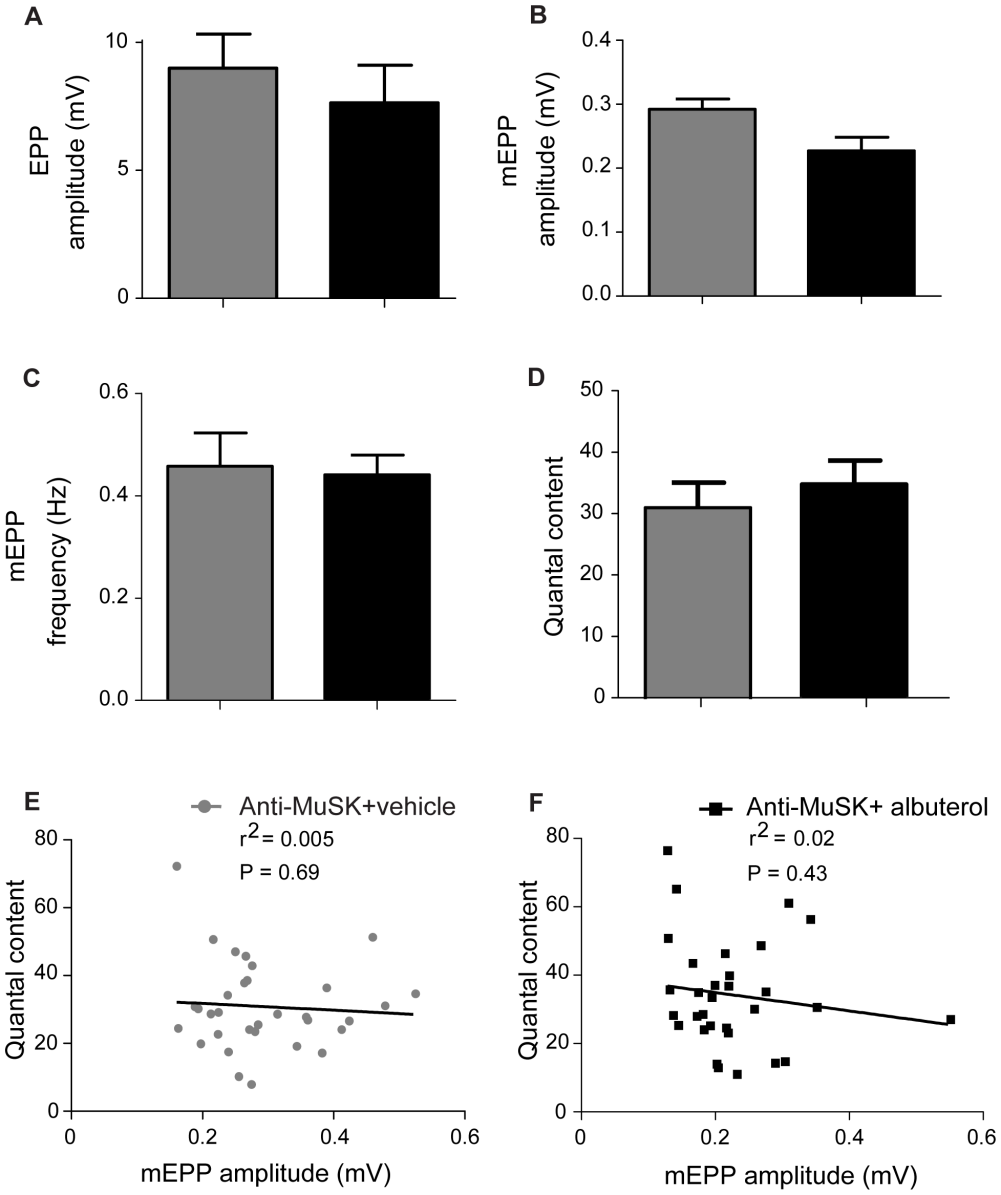
Albuterol did not improve endplate potentials in mice injected with anti-MuSK IgG. Comparison of endplate potentials (EPPs) for myasthenic mice treated with either 8 mg/kg/day albuterol or vehicle. Recordings were made from the phrenic nerve-diaphragm muscle preparation on day 15 of the anti-MuSK IgG injection series. (**A**) Amplitude of nerve-evoked EPPs from mice injected with anti-MuSK IgG and treated with albuterol (8 mg/kg/day; black bar) or vehicle (grey bar). (**B**) Amplitudes of spontaneous mEPPs from the same mice. (**C**) Frequency of mEPPs. (**D**) Quantal content. Data in panels A–D represent the mean ± SEM for n = 3 mice in each treatment group (unpaired Student’s t-test). None of these parameters differed significantly between albuterol- and vehicle-treatment groups. (**E**) Scatter plot of quantal content versus mEPP amplitude for endplates in the diaphragm muscle of anti-MuSK-injected mice that were treated with vehicle (each endplate is represented by a filled circle). Data were fitted by linear regression. No significant correlation was found (P = 0.69). Probability values (P) reflect the likelihood that the slope was non-zero. (**F**) Quantal content versus mEPP amplitude for endplates of anti-MuSK-injected mice that were treated with albuterol (P = 0.43).

The phrenic nerve-hemidiaphragm muscle preparation from the same mice was then used to record endplate potentials. As expected, the amplitudes of nerve-evoked EPPs and spontaneous mEPPs were rather low in anti-MuSK injected mice (∼0.3 mV), compared to values previously recorded in healthy mice (∼0.6 mV; [31], [38]). For mice injected with anti-MuSK IgG, treatment with albuterol (8 mg/kg/day) did not significantly change the amplitudes of the EPPs or mEPPs (Fig. 5A & B), the frequency of spontaneous mEPPs (Fig. 5C), nor the number of quanta released per nerve impulse (quantal content; Fig. 5D). Among NMJs there is normally an inverse relationship between quantal content and mEPP amplitude [49], [50]. This relationship is thought to reflect the long-term adaptation of presynaptic transmitter release to changes in postsynaptic responsiveness to acetylcholine. The inverse relationship failed in models of anti-MuSK-positive MG (Fig. 5E; [31], [37], [38]). Albuterol treatment did not restore a significant inverse relationship between quantal content and mEPP amplitude (Fig. 5F). In summary, albuterol treatment did not significantly improve neuromuscular transmission at the endplates studied.

### Influence of Anti-MuSK IgG and Albuterol upon Muscle Fiber Cross-sectional Area

**Figure 6:**
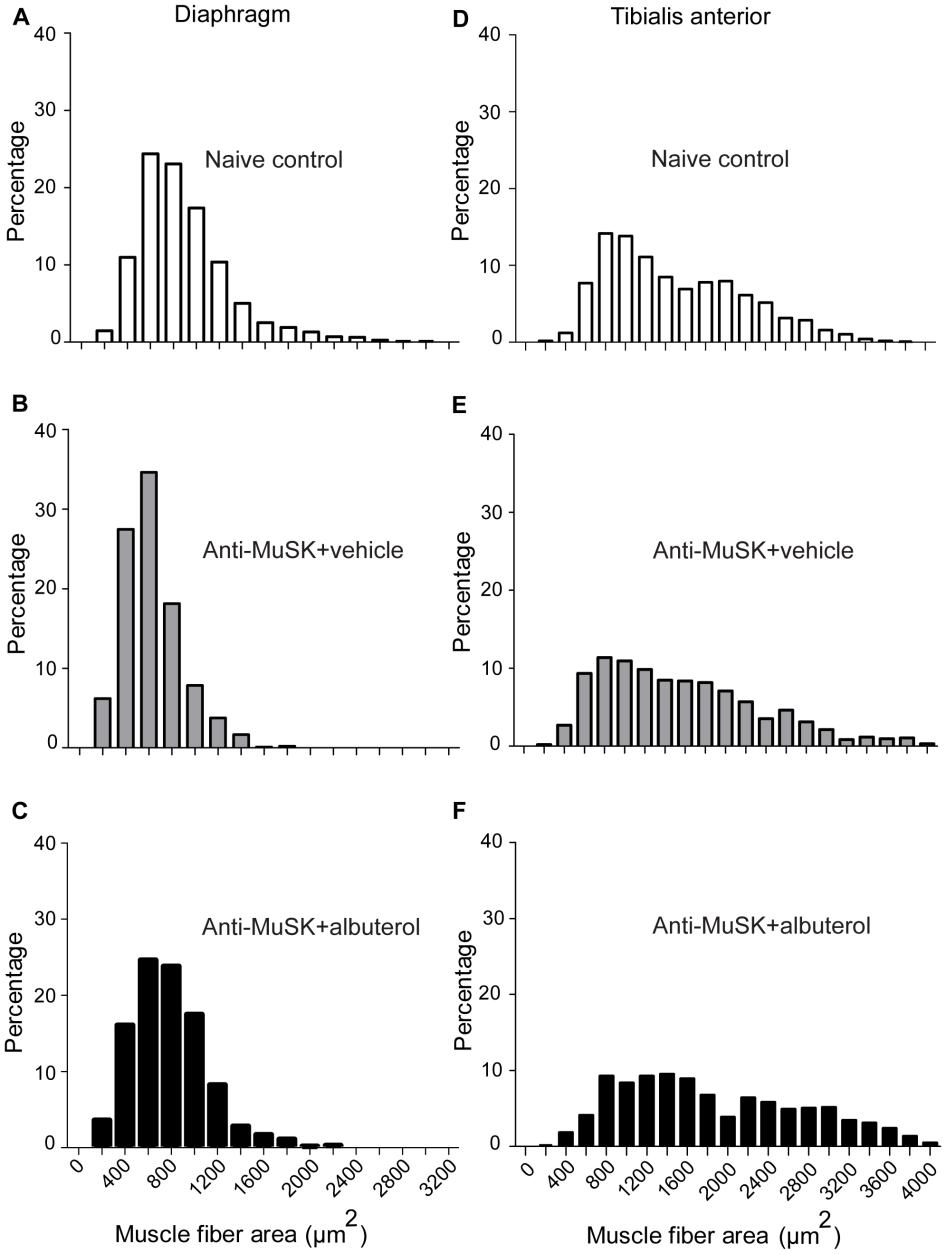
Cross-sectional area of muscle fibers. (**A–C**) Frequency distributions of muscle fiber cross-sectional areas for the diaphragm muscle. (**D–F**) Cross-sectional areas for fibers from the tibialis anterior muscle. Data were pooled from healthy naive mice (A & D; n = 5 mice) or mice that received 15 daily injections of anti-MuSK IgG together with vehicle (B & E; n = 4 mice) or 8 mg/kg/day albuterol (C & F; n = 4 mice).

Since β2-adrenoceptor agonists can cause muscle fiber hypertrophy we measured the cross-sectional area of muscle fibers in the mice studied above. The cross-sectional area of fibers in the diaphragm muscle followed an approximate normal distribution (Fig. 6A) with a mean of 880±86 µm^2^ (mean ± SEM; n = 5 mice). Injection of anti-MuSK IgG significantly reduced the mean cross-sectional area of diaphragm muscle fibers to 620±43 µm^2^ (n = 4 mice) when compared with healthy naive mice (P = 0.045; two-tailed Student t-test; Fig. 6B). In the diaphragm muscle of albuterol-treated mice an upward trend in fiber cross-sectional area (780±72 µm^2^; n = 4 mice; Fig. 6C), compared to vehicle-treated mice, did not reach significance (P = 0.12). In the tibialis anterior muscle of naive mice, the distribution of fiber cross-sectional areas appeared to be bi-modal (Fig. 6D) with a median of 1344 µm^2^. In mice that received anti-MuSK IgG plus vehicle the median cross-sectional area (1563 µm^2^) was not significantly different to naive mice (P = 0.36; one-tailed Mann-Whitney test). In the tibialis anterior muscle of albuterol-treated mice an increase in median fiber cross sectional area (1859 µm^2^) compared to vehicle treated-mice did not reach significance (P = 0.17; Fig. 6E & F).

## Discussion

In our passive IgG-transfer mouse model of anti-MuSK MG albuterol treatment reduced the degree of whole-body weakness and weight loss. However this clinically positive result with a cheap, licensed and relatively safe drug could not be well explained by the neuromuscular measures we examined. Albuterol did not alter the autoantibody-induced loss of postsynaptic AChR, nor did albuterol improve electrophysiological measures of synaptic function. Mice treated with albuterol (8 mg/kg/day) throughout the two-week anti-MuSK IgG injection series showed areas and intensities of endplate AChR staining as well as EPP amplitudes that were similar to vehicle-treated myasthenic mice. The primary pathogenic mechanism of anti-MuSK IgG in rodent models seems to be a decline in endplate AChR density/number [32], [33], [34], [35], [41] with a consequent reduction in quantal amplitude [36], [37], [38], [40]. Albuterol did not inhibit these primary measures of synaptic impairment in the passive IgG transfer model of anti-MuSK MG. However, some features of endplate integrity were significantly better with albuterol, suggesting the need for further studies in other animal models.

Long-term treatment with albuterol has been successful in improving strength for patients with forms of congenital myasthenia that have common features with anti-MuSK MG. These include patients with mutations in the ColQ and Dok-7 genes [14], [15], [16], . Dok-7 forms a complex with the cytoplasmic domain of MuSK that is important for MuSK kinase activation [10]. ColQ tethers MuSK to acetylcholinesterase in the synaptic cleft [51]. Thus mutations in either the ColQ or Dok-7 genes might be expected to impair MuSK signaling at the endplate. Since albuterol and ephedrine were beneficial in treating these congenital disorders we reasoned that these drugs might also be beneficial in anti-MuSK MG. Protein kinase A (PKA; a downstream effector of β2-adrenoceptors) is known to bind to rapsyn (a downstream effector of MuSK). Both rapsyn and PKA help to stabilize AChRs within the postsynaptic membrane [52], [53]. Thus our working hypothesis was that activation of muscle β2-adrenoceptors might compensate for reduced MuSK/rapsyn function in the postsynaptic membrane, thereby sparing the postsynaptic AChR clusters [18]. Our results in the passive IgG transfer model provide little support for this hypothesis.

Loss of postsynaptic AChR appears to be a primary cause of synaptic failure in animal models of anti-MuSK MG [32], [33], [34], [35], [36], [37], [38], [40], [41], [54], [55]. For the endplates we studied, albuterol did not prevent anti-MuSK-induced reductions in the intensity or area of AChRs, nor did albuterol enhance synaptic transmission. Compound muscle action potentials in the gastrocnemius muscle of anaesthetized mice, and *ex vivo* EPP recordings from the diaphragm revealed no functional improvement in those anti-MuSK-injected mice that were treated with albuterol. In the diaphragm muscle of mice that became weak after injections of anti-MuSK IgG there were reductions in the number of endplates per photomicrograph, suggesting the complete loss of many NMJs [41]. In the present study we found a comparable reduction in counts of the number of endplates. Albuterol treatment was unable to prevent the apparent loss of endplates from the diaphragm muscle.

Several caveats need to be considered in assessing the results. Our passive IgG transfer protocol involved injecting mice with large amounts of patient IgG (25 mg/day for 15 days). This model yields a robust and reproducible onset of synaptic failure and myasthenic weakness within two weeks [33],[38]. However the relatively rapid onset of synaptic impairment and weakness may not perfectly reflect the chronic disease state in patients. Moreover our mouse model does not mimic the congenital (Dok-7 or ColQ) myasthenias where clear strength gains have been reported in patients following treatment with albuterol or ephedrine. By reducing AChR cluster fragmentation (Fig. 3D) it is possible that over long-term treatment of patients [18], [56], albuterol might enhance postsynaptic AChR density. The mode of delivery of albuterol might also influence its efficacy. Congenital myasthenia patients received albuterol in pill form while our mice were continuously infused with albuterol. Continuous delivery of albuterol led to β2-adrenoceptor loss and desensitization in lung tissue [57]. Perhaps pulsatile treatment might cause less desensitization. Notwithstanding these considerations, the present findings illustrate that improvement in whole-body weakness following albuterol treatment does not necessarily imply a measurable reversal of neuromuscular transmission failure.

By what other mechanism/s might albuterol have reduced weakness in our anti-MuSK injected mice? Firstly, it remains possible that albuterol positively influenced aspects of synaptic function other than those that we have measured. It is also conceivable that albuterol might enhance neural drive to myasthenic muscles. However, in healthy rodents albuterol was found to reduce spontanous locomotor activity, rather than increasing mobility [58], [59]. On the other hand, activation of the β2-adrenceptor acutely increased tetanic force in rodent muscles by about 15% [60]. In fast-twitch mouse muscle this involved a PKA-mediated phosphorylation of the ryanodine receptor that potentiated calcium release from the sarcoplasmic reticulum [61]. This mechanism might have helped enhance strength in our albuterol-treated myasthenic mice, although its significance for humans has been questioned [62]. Albuterol might also have helped spare weakness by inhibiting muscle atrophy. After 14 daily injections of anti-MuSK-positive patient IgG the cross-sectional area of diaphragm muscle fibers was reduced by 29% compared to naive control mice (Fig. 6A&B). This fiber atrophy might have been an indirect result of impaired neuromuscular transmission [31], [38]. Activation of skeletal muscle β2-adrenceptors can drive protein synthesis, inhibit protein catabolism, and produce fiber hypertrophy via an emerging network of signalling pathways [63], [64]. Indeed, albuterol treatment was associated with a trend towards increased muscle fiber cross-sectional area in anti-MuSK injected mice (Fig. 6). While this effect did not reach statistical significance, there is good theoretical reason to think that albuterol will inhibit atrophy and/or cause hypertrophy to enhance the force produced by those fibers still under the control of the motor nerve.

While albuterol did not improve the function of the NMJs in our recordings, it did produce significant improvements to two out of eight measures of NMJ integrity in anti-MuSK-injected mice. Anti-MuSK injections cause the break-up of the large postsynaptic AChR plaque into multiple small (fragmented) AChR clusters. Albuterol reduced the extent of AChR cluster fragmentation. Secondly, in albuterol-treated mice a higher proportion of the surviving endplate AChRs retained coverage by nerve terminal staining, suggesting more-complete innervation. These two positive effects of albuterol lay the groundwork for further studies in other animal models of myasthenias where the onslaught might be more gradual and the subtle beneficial effects of albuterol may improve the function of the myasthenic NMJ.
